# Quality indicators in continuous renal replacement therapy (CRRT) care in critically ill patients: protocol for a systematic review

**DOI:** 10.1186/s13643-015-0088-1

**Published:** 2015-07-30

**Authors:** Oleksa Rewa, Pierre-Marc Villeneuve, Dean T. Eurich, Henry T Stelfox, RT Noel Gibney, Lisa Hartling, Robin Featherstone, Sean M Bagshaw

**Affiliations:** Division of Critical Care Medicine, Faculty of Medicine and Dentistry, University of Alberta, 8440 112 St. NW, Critical Care Medicine 2-124E Clinical Sciences Building, Edmonton, Alberta T6G 2B7 Canada; 2-040 Li Ka Shing Center for Health Research Innovation, School of Public Health, University of Alberta, Edmonton, Alberta Canada; Department of Critical Care Medicine, University of Calgary, Calgary, Alberta Canada; Department of Pediatrics, Faculty of Medicine and Dentistry, Aberhart Centre, Room 8417, Edmonton, Alberta T6G 1Z1 Canada; Alberta Research Center for Health Evidence (ARCHE), University of Alberta, 4-486D Edmonton Clinic Health Academy, 11405 – 87 Avenue, Edmonton, Alberta T6G 1C9 Canada

**Keywords:** Quality indicator, Effectiveness, Continuous renal replacement therapy, Dialysis, Critical care, Intensive care

## Abstract

**Background:**

Renal replacement therapy is increasingly utilized in the intensive care unit (ICU), of which continuous renal replacement therapy (CRRT) is most common. Despite CRRT being a relatively resource-intensive and expensive technology, there remains wide practice variation in its application. This systematic review will appraise the evidence for quality indicators (QIs) of CRRT care in critically ill patients.

**Methods:**

Ovid MEDLINE, Ovid EMBASE, CINAHL, and the Cochrane Library including the Cochrane Database of Systematic Reviews, the Cochrane Central Register of Controlled Trials (CENTRAL), and databases from the National Information Center of Health Services Research and Health Care Technology will be searched for original studies involving QIs in CRRT. Gray literature sources will be searched for technical reports, practice guidelines, and conference proceedings. Websites of relevant organizations will be identified, and industry leaders in the development and marketing of CRRT technology and non-profit organizations that represent key opinion leads in the use of CRRT will be contacted. We will search the Agency of Healthcare Research and Quality National Quality Measures Clearinghouse for CRRT-related QIs. Studies will be included if they contain quality measures, occur in critically ill patients, and are associated with CRRT. Analysis will be primarily descriptive. Each QI will be evaluated for importance, scientific acceptability, usability, and feasibility using the four criteria proposed by the United States Strategic Framework Board for a National Quality Measurement and Reporting System. Finally, QIs will be appraised for their potential operational characteristics, for their potential to be integrated into electronic medical records, and on their affordability, if applicable.

**Discussion:**

This systematic review will comprehensively identify and synthesize QIs in CRRT. The results of this study will fuel the development of an inventory of essential QIs to support the appropriate, safe, and efficient delivery of CRRT in critically ill patients.

**Systematic review registration:**

PROSPERO CRD42015015530.

**Electronic supplementary material:**

The online version of this article (doi:10.1186/s13643-015-0088-1) contains supplementary material, which is available to authorized users.

## Background

Acute renal replacement therapy (RRT) is used in 8–10 % of critically ill patients, to support injured or overtly failing kidneys in the context of multiple organ dysfunction syndrome [1–4]. RRT utilization is increasing steadily [2–5]. Population-based estimates have suggested the incidence of acute RRT has increased by greater than 10 % per year over the past decade and continuous renal replacement therapy (CRRT) remains the most common form of RRT used in intensive care unit (ICU) settings [6–8]. While CRRT has not shown a clear survival benefit over conventional intermittent forms of RRT in critically ill patients, [9–11] recent data have shown initial therapy with CRRT may be associated with improved long-term recovery of kidney function [12, 13]. These observations imply the utilization of CRRT will continue to increase.

CRRT is a continuous method of blood purification that theoretically provides slow uninterrupted clearance of retained endogenous and exogenous toxins, along with providing acid-base, electrolyte, and volume homeostasis. While CRRT is intended to function 24 h a day (analogous to a native kidney), it is often interrupted [14, 15]. Unplanned treatment interruption can negatively impact its efficiency and safety [14]. Recent trials have shown lower dose-intensive CRRT (25 ml/Kg/h) is as effective as higher dose-intensive (40 ml/Kg/h) CRRT on outcomes, [1] a view supported by the Kidney Disease: Improving Global Outcomes (KDIGO) Clinical Practice Guidelines for Acute Kidney Injury [5]. However, there remains important disparity in practice between the prescribed and delivered dose in CRRT [6]. Many additional aspects of CRRT in critically ill patients remain uncertain, in particular the ideal circumstances and optimal timing for when to initiate CRRT [9, 11]. This again contributes to heterogeneity in the practice and delivery of suboptimal quality CRRT care [7, 8, 16, 17]. These issues can be broadly classified into potential quality domains related to the prescription and delivery of CRRT (Table 1).

**Table 1:** Summary of potential quality indicator themes and measures

Themes	Measures
Dose prescription	High vs. low dose
Dose delivery	Percentage of prescribed dose delivered
Anticoagulation selection	Heparin vs. citrate vs. none
Anticoagulation monitoring	PTT monitoring, citrate monitoring
Anticoagulation complications	Bleeding, hypocalcaemia, incidence of HIT
Treatment interruption	Number of interruptions and duration of interruptions; time to establish new circuit
Catheter-related issues	Infections, bleeding, obstruction/thrombosis
Circuit-related issues	Filter clotting, pressure alarming

*HIT* heparin-induced thrombocytopenia

While CRRT is generally a resource-intensive and expensive technology [3, 10, 18], it remains the default modality of support most frequently used for severely ill patients at high risk for death [1, 8, 12, 13]. Practice variation in utilization of CRRT has been shown to independently contribute to higher risk for less favorable outcomes and itself is considered a measure of poor quality care [14, 15, 19]. While this variation may stem from important knowledge gaps in evidence to guide best practice, different providers (e.g., nephrology vs. intensive care) and limited provider and institutional expertise in CRRT, coupled with a paucity of clearly defined quality measures of CRRT care, are likely also important contributors. To date, no study has systematically mapped or evaluated the scope of quality measures in CRRT care.

Accordingly, we will perform a systematic review of quality indicators (QIs) of CRRT care. This is a critical initial step to reduce low-quality CRRT care, optimize resource utilization, and improve outcomes. We believe our review will map important themes in CRRT care to identify and close “evidence care gaps” through better monitoring, reporting, benchmarking, and process reassessment.

## Methods

### Study design

We will perform a systematic review to identify and evaluate QIs for the prescription, delivery, and monitoring and their association with patient-centered and health economic outcomes (if available) for critically ill patients receiving CRRT using the guidelines from Cochrane and Center for Reviews and Dissemination and described according to the PRISMA-P guideline (Additional file 1) [20–22].

### Study registration

This systematic review is registered with PROSPERO (CRD42015015530).

### Criteria for considering studies for this review

#### Inclusion criteria

Studies will be included if they mention *all* of the following themes: (1) *quality measure*, i.e., intended to evaluate the care received by patients treated with CRRT; (2) *intensive care*, i.e., intended to refer to patients (adults, children, and neonates) supported in an intensive care unit setting; (3) *continuous renal replacement therapy*, i.e., the prescription, delivery, or outcome associated with CRRT; (4) language of study being English, French, German, Italian, or Spanish; (5) publication after 1990; and (6) levels of evidence, all primary studies (i.e., randomized control trials, cohort studies, case-control studies, case series, and qualitative or mixed methods studies), secondary analyses, or evidence syntheses (i.e., systematic reviews, meta-analyses, and Cochrane reviews), as well as targeted gray literature including technical reports from industry or to governments or health care agencies. These studies will not be limited to comparative studies and will include any literature with mention of QIs. An initial screening of retrieved literature considered drug monitoring and drug levels as a potential QI; however, given the extensive number of citations related to this theme, we believed this would be ideally suited to a separate dedicated study and omitted is from this systematic review.

#### Exclusion criteria

Studies will be excluded that do not fulfill all of the above criteria.

#### Search methods for identification of studies

The search strategy will be developed in consultation with an information specialist at the Alberta Research Centre for Health Evidence (ARCHE) at the University of Alberta and will be peer-reviewed by another librarian [23]. The information specialist will search electronic databases: Ovid MEDLINE, Ovid EMBASE, Cumulative Index to Nursing and Allied Health Literature (CINAHL) via EBSCO host, and the Cochrane Library including the Cochrane Database of Systematic Reviews and the Cochrane Central Register of Controlled Trials (CENTRAL). In addition, databases from the National Information Center of Health Services Research and Health Care Technology will be searched. A combination of the following search themes will be used: (1) continuous renal replacement therapy, hemofiltration, hemodialfiltration, dialysis, renal replacement therapy, and renal support and (2) intensive care, critical care, critical illness, multi-organ dysfunction, and multi-organ failure (see Table 2). Results will be limited to human studies, published in English, French, German, Italian, or Spanish since 1990. Bibliographic records will be exported to an EndNote X7 (Thomson Reuters, Philadelphia, Pennsylvania) database for screening.

**Table 2:** The strategy will be adapted and executed in the above databases for the full search: Ovid MEDLINE, CINAHL@ VIA EBSCOHOST, EMBASE@ VIA OBID, AND Cochrane Library

1.	Acute Kidney Injury/th
2.	Hemodiafiltration/
3.	Renal Dialysis/
4.	Renal Replacement Therapy/
5.	(dialys* or hemodialys* or haemodialys*).tw,kf.
6.	(haemodiafiltrat* or haemo diafiltrat* or haemofiltrat* or haemo filtrat* or hemodiafiltrat* or hemo diafiltrat* or hemofiltrat* or hemo filtrat*).tw,kf.
7.	(renal replacement adj2 (therap* or treatm* or support*)).tw,kf.
8.	RRT.tw,kf.
9.	or/1-8
10.	(24h or 24hr* or 24 hour* or 24 hr* or continual* or continuous* or twenty four hour* or twenty four hr* or twentyfour hour* or twentyfour hr*).mp.
11.	and/9-10
12.	CRRT.tw,kf.
13.	or/11-12
14.	Critical Care/
15.	Critical Illness/
16.	exp Intensive Care/
17.	Intensive Care Units/
18.	exp Intensive Care Units, Pediatric/
19.	Multiple Organ Failure/
20.	critical care.tw,kf.
21.	critical* ill*.tw,kf.
22.	(ICU* or NICU* or PICU*).tw,kf.
23.	intensive care.tw,kf.
24.	intensivist*.tw,kf.
25.	(multi* organ adj (disfunction* or dis function* or dysfunction* or dys function* or failure*)).tw,kf.
26.	(multi* system adj (disfunction* or dis function* or dysfunction* or dys function* or failure*)).tw,kf.
27.	or/14-26
28.	and/13,27
29.	animals/ not (animals/ and humans/)
30.	28 not 29
31.	limit 30 to (english or french or german or italian or spanish)
32.	limit 31 to yr="1990-Current"
33.	remove duplicates from 32

Additional sources will be included in the search strategy. The cited and citing references of selected key studies will be searched for relevant articles. Gray literature sources will be searched for technical reports, practice guidelines, and conference proceedings. We will identify and search the websites of relevant organizations (i.e., Canadian Society of Nephrology, European Societies of Nephrology [ERA-EDTA], National Kidney Foundation, American Society of Nephrology, American Society for Artificial Internal Organs, European Society for Artificial Organs). Industry leaders in the development and marketing of CRRT technology (i.e., Baxter-Gambro Renal Inc., NxStage Inc., Fresenius Medical Care Inc., Bellco Inc., Medica Inc.) will be contacted. Non-profit organizations that represent key opinion leads in critical care nephrology and the use of CRRT (i.e., Acute Dialysis Quality Initiative) will also be contacted. We will search the Agency of Healthcare Research and Quality National Quality Measures Clearinghouse (www.qualitymeasures.ahrq.gov) for CRRT-related quality measures. Finally, we will survey an inter-disciplinary group of knowledge users, clinical experts, and decision-makers (i.e., physicians, nurses, engineers) experienced with the provision of CRRT in critically ill patients to elicit additional potential quality measures.

#### Data extraction and analysis

Eligible articles will be identified through two phases. In the first phase, two authors will independently review the titles and abstracts of all retrieved articles and documents using EndNote X7 (Thomson Reuters, Philadelphia, Pennsylvania) for potential inclusion into the systematic review. Disagreements will be resolved through discussion. In the case of unresolved matters, a third party will be involved. In the second phase, full texts of the selected articles will be retrieved and information abstracted using standardized forms. The same two authors will conduct this independently. Abstracted data will be then compared amongst the two authors, and disagreements will also be resolved through discussion. In the case of unresolved matters, a third party will be involved. The authors of the retrieved studies and/or documents will be contacted for further information if necessary. Methodological quality will be rated using the Newcastle-Ottawa Quality Assessment Scale (NOS) for observational studies and a modified version of BOAS for before-after studies, as applicable [24]. Qualitative studies will be evaluated using the COnsensus-based Standards for the selection of health Measurement INstruments (COSMIN checklist) with four-point scale [25].

QIs will be identified from included articles and documents and from the survey of experts and key stakeholders. Two independent authors will collect data on the properties of measurement and characteristics of each of the identified QIs. The relevance of each QI will then be evaluated using the four criteria proposed by the United States Strategic Framework Board for a National Quality Measurement and Reporting System (importance, scientific acceptability, usability, and feasibility) [26]. Importance will be based on how each QI may inform about CRRT prescription, delivery, and monitoring and association with patient-centered and health economic outcomes. Scientific acceptability will assess how plausible each QI measures attributes of CRRT and outcomes. Usability and feasibility will characterize the logistics and process of implementation of each QI into clinical practice. These outcomes will be further evaluated in the second phase of this project when the evidence base for each QI will be evaluated and ranked by key knowledge users, stakeholders, and experts. Candidate QIs will be each evaluated for their operational characteristics such as association with circuit lifespan, resource intensity (i.e., nursing workload), and health care costs, as well as for their potential to be integrated into electronic medical records, if applicable.

### Analysis

Descriptive analyses will be performed on all articles and QIs. Each QI will be categorized first according to the structure, process, and outcome framework and then by agreed upon domains of evaluation. The Donabedian framework for examining health services and evaluating quality of care, along with the identified relevant domains of evaluation, will be used and modified as the models and frameworks are identified. Due to the anticipated heterogeneity of QIs and methods of ascertainment, a comprehensive inventory of QIs will be developed and summarized as counts and proportions. These summary counts and proportions will be further stratified based on relevant features such as study design, domains of health care quality, rank, and domains of evidence and evaluated using chi-square tests. When possible, articles and QIs will be pooled and further analysis will be performed; however, due to the heterogeneity as well as broad scope of material, it is expected that it will not be possible to pool all QIs for analysis. All analyses will be performed using STATA statistical software, version 13 (StataCorp, College Station, Texas).

## Discussion

CRRT is the predominant form of acute RRT provided to critically ill patients, and its utilization is increasing. CRRT is a complex technology that is resource intensive, costly, and requires specialized training by health providers and is susceptible to treatment error.

There is considerable practice variation in CRRT care. CRRT can be prescribed and delivered by either or both nephrology and/or intensive care [27]. To date, we are unaware of any prior comprehensive and rigorous evaluation of QIs in CRRT care. In our view, given the complexity, cost, and resource intensiveness of CRRT implementation, this is a critical knowledge gap in the delivery of one of the core life support technologies that define intensive care. This systematic review will establish an inventory of potential CRRT-specific QIs that will provide knowledge users, clinicians, administrators, and researchers with robust measures to continuously appraise the quality, safety, and effectiveness of CRRT care. Moreover, these QIs may present opportunities for further innovation in CRRT care, contribute to improve patients’ outcomes, and better utilization of health resources. We believe that this systemic review is timely and will make a valuable contribution by helping to identify and address current existing evidence care gaps. Moreover, our systematic review will lead to future opportunities to establish a research agenda that will continue to address deficiencies in our knowledge surrounding QIs in the delivery of CRRT.

From our systematic review, the next steps in our program will involve an evaluation of each identified CRRT QI by key knowledge users, stakeholders, and experts. QIs will be ranked using a Delphi process to develop a prioritized consensus inventory of relevant CRRT QIs across the spectrum of CRRT care for implementation into clinical practice. We anticipate the findings from our review and this consensus process will inform broader implementation of quality measures in CRRT care and be integrated into educational and/or training programs to support safe and effective CRRT care for critically ill patients.

### Expected limitations

It is anticipated that due to the paucity of focused literature on QIs in CRRT care, the scope of QIs will show considerable heterogeneity across a spectrum of scientific rigor and relevance. The comparisons across strata of QIs are likely to be underpowered in chi-squared analysis owing to the anticipated heterogeneity across measures; however, such analysis is not the primary objective of the review. It is also anticipated that some of the QIs will be significant and high quality while others will be poorer quality. In addition, we have limited our search strategy to include only studies published in selected languages (English, French, German, Italian, or Spanish). We recognize this may result in omission of studies describing potential QIs; however, we believe these languages will capture the majority of high-quality published research in CRRT. We will utilize the NOS or COSMIN checklist to quantify and evaluate the risk of bias across studies, and these measures will be included in our analysis. Finally, it is expected that there will be limited evidence of impact of adoption of individual or combinations of QIs in CRRT programs.

## Additional file

Additional file 1:
**Preferred Reporting Items for Systematic review and Meta-Analysis Protocols (PRISMA-P) 2015 checklist: recommended items to address in a systematic review protocol.**

## Abbreviations

CRRT: continuous renal replacement therapy
ICU: intensive care unit
QI: quality indicator
RRT: renal replacement therapy

## References

[1] Bellomo R, Cass A, Cole L, Finfer S, Gallagher M, RENAL Replacement Therapy Study Investigators (2009). Intensity of continuous renal-replacement therapy in critically ill patients. N Engl J Med.

[2] Siddiqui NF, Coca SG, Devereaux PJ, Jain AK, Li L, Luo J (2012). Secular trends in acute dialysis after elective major surgery--1995 to 2009. Can Med Assoc J.

[3] Rewa O, Bagshaw SM (2014). Acute kidney injury—epidemiology, outcomes and economics. Nat Rev Nephrol.

[4] Hsu RK, McCulloch CE, Dudley RA, Lo LJ, Hsu C-Y (2013). Temporal changes in incidence of dialysis-requiring AKI. J Am Soc Nephrol.

[5] Kellum JA, Lameire N (2012). KDIGO clinical practice guideline for acute kidney injury. Kidney Int Suppl.

[6] Lyndon WD, Wille KM, Tolwani AJ (2012). Solute clearance in CRRT: prescribed dose versus actual delivered dose. Nephrol Dial Transplant.

[7] Prowle JR, Bellomo R (2010). Continuous renal replacement therapy: recent advances and future research. Nat Rev Nephrol.

[8] Uchino S, Bellomo R, Morimatsu H, Morgera S, Schetz M, Tan I (2007). Continuous renal replacement therapy: a worldwide practice survey. The beginning and ending supportive therapy for the kidney (B.E.S.T. kidney) investigators. Intensive Care Med.

[9] Karvellas CJ, Farhat MR, Sajjad I, Mogensen SS, Leung AA, Wald R (2011). A comparison of early versus late initiation of renal replacement therapy in critically ill patients with acute kidney injury: a systematic review and meta-analysis. Crit Care.

[10] Bagshaw SM, Berthiaume LR, Delaney A, Bellomo R (2008). Continuous versus intermittent renal replacement therapy for critically ill patients with acute kidney injury: A meta-analysis*. Crit Care Med.

[11] Bagshaw SM, Uchino S, Bellomo R, Morimatsu H, Morgera S, Schetz M, Beginning and Ending Supportive Therapy for the Kidney BEST Kidney Investigators (2009). Timing of renal replacement therapy and clinical outcomes in critically ill patients with severe acute kidney injury. J Crit Care.

[12] Schneider AG, Bellomo R, Bagshaw SM, Glassford NJ, Lo S, Jun M (2013). Choice of renal replacement therapy modality and dialysis dependence after acute kidney injury: a systematic review and meta-analysis. Intensive Care Med.

[13] Wald R, Shariff SZ, Adhikari NKJ, Bagshaw SM, Burns KEA, Friedrich JO (2014). The association between renal replacement therapy modality and long-term outcomes among critically ill adults with acute kidney injury. Crit Care Med.

[14] Uchino S, Fealy N, Baldwin I, Morimatsu H, Bellomo R (2003). Continuous is not continuous: the incidence and impact of circuit “down-time” on uraemic control during continuous veno-venous haemofiltration. Intensive Care Med.

[15] Tolwani A (2012). Continuous renal-replacement therapy for acute kidney injury. N Engl J Med.

[16] Overberger P, Pesacreta M, Palevsky PM, VA/NIH Acute Renal Failure Trial Network (2007). Management of renal replacement therapy in acute kidney injury: a survey of practitioner prescribing practices. Clin J Am Soc Nephrol.

[17] Ricci Z, Ronco C, D’amico G, De Felice R, Rossi S, Bolgan I (2006). Practice patterns in the management of acute renal failure in the critically ill patient: an international survey. Nephrol Dial Transplant.

[18] Manns B, Doig CJ, Lee H, Dean S, Tonelli M, Johnson D (2003). Cost of acute renal failure requiring dialysis in the intensive care unit: clinical and resource implications of renal recovery*. Crit Care Med.

[19] Elseviers MM, Lins RL, Van der Niepen P, Hoste E, Malbrain ML, Damas P, SHARF investigators (2010). Renal replacement therapy is an independent risk factor for mortality in critically ill patients with acute kidney injury. Crit Care.

[20] Higgins JP, Green S (Editors). Cochrane Handbook for Systemaic Reviews of Interventions (version. 5.1). The Cochrane Collections, 2011. Available from: www.cochrane-handbook.org. (Accessed September 22, 2014).

[21] Centre for Reviews and Dissemination (2009). Systematic reviews: CRD’s guidance for undertaking reviews in health care.

[22] Moher D, Shamseer L, Clarke M, Ghersi D, Liberati A, Petticrew M, PRISMA-P Group (2015). Preferred reporting items for systematic review and meta-analysis protocols (PRISMA-P) 2015 statement. Syst Rev.

[23] Sampson M, McGowan J, Cogo E, Grimshaw J, Moher D, Lefebvre C (2009). An evidence-based practice guideline for the peer review of electronic search strategies. J Clin Epidemiol.

[24] Wells GA, Shea B, O’Connell D, Peterson J, Welch V, Losos M, Tugwell P (2011). The Newcastle-Ottawa Scale (NOS) for assessing the quality if nonrandomized studies in meta-analyses.

[25] Dorfman TL, Sumamo Schellenberg E, Rempel GR, Scott SD, Hartling L (2014). An evaluation of instruments for scoring physiological and behavioral cues of pain, non-pain related distress, and adequacy of analgesia and sedation in pediatric mechanically ventilated patients: a systematic review. Int J Nurs Stud.

[26] McGlynn EA (2003). Introduction and overview of the conceptual framework for a national quality measurement and reporting system. Med Care.

[27] Pannu N, Klarenbach S, Wiebe N, Manns B, Tonelli M, Network for the Alberta Kidney Disease (2008). Renal replacement therapy in patients with acute renal failure: a systematic review. JAMA.

